# Factors Associated with Long-Term Use of Hypnotics among Patients with Chronic Insomnia

**DOI:** 10.1371/journal.pone.0113753

**Published:** 2014-11-19

**Authors:** Yoshikazu Takaesu, Yoko Komada, Shoichi Asaoka, Tatsuo Kagimura, Yuichi Inoue

**Affiliations:** 1 Department of Psychiatry, Tokyo Medical University, 6-7-1 Nishishinjuku, Shinjuku-ku, Tokyo, 160-0023, Japan; 2 Department of Somnology, Tokyo Medical University, 6-7-1 Nishishinjuku, Shinjuku-ku, Tokyo, 160-0023, Japan; 3 Japan Somnology Center, Neuropsychiatric Research Institute, 1-24-10 Yoyogi, Shibuya-ku, Tokyo, 151-0053, Japan; 4 Translational Research Informatics Center, Foundation for Biomedical Research and Innovation, 1-5-4 Minatojima-minamimachi, Chuo-ku, Kobe, Hyogo, 650-0047, Japan; University of Rochester, United States of America

## Abstract

This study investigated factors associated with long-term use of benzodiazepines (BZDs) or benzodiazepine receptor agonists (BzRAs) as hypnotics in patients with chronic insomnia. Consecutive patients (n = 140) with chronic insomnia were enrolled in this study (68 men and 72 women; mean age, 53.8±10.8 years). All patients filled out a self-assessment questionnaire asking clinical descriptive variables at the baseline of the treatment period; patients received the usual dose of a single type of BZD or BzRA. The Pittsburgh Sleep Quality Index (PSQI) and the Zung Self-Rating Depression Scale were self-assessed at the baseline, and the former was re-evaluated at the time of cessation of medication or at the end of the 6-month treatment period. The PSQI included the following sub-items: evaluating sleep quality (C1), sleep latency (C2), sleep duration (C3), habitual sleep efficiency (C4), frequency of sleep disturbance (C5), use of sleeping medication (C6), and daytime dysfunction (C7). Among the patients, 54.6% needed to continue hypnotics for a 6-month treatment period. Logistic regression analysis revealed that, among descriptive variables, only the PSQI score appeared as a significant factor associated with long-term use {odds ratio (OR) = 2.8, 95% confidence interval (CI) = 2.0–4.0}. The receiver operating curve (ROC) analysis identified that the cut-off PSQI total score at the baseline for predicting long-term use was estimated at 13.5 points (area under the curve = 0.86, 95% CI = 0.8–0.92). Among the sub-items of PSQI, the increases in C1: (OR = 8.4, 95% CI = 2.4–30.0), C3: (OR = 3.6, 95% CI = 1.1–11.5), C4: (OR = 11.1, 95% CI = 3.6–33.9), and C6: (OR = 3.4, 95% CI = 1.9–6.2) scores were associated with long-term use. This study revealed that a high PSQI score at the baseline, particularly in the sub-items relating to sleep maintenance disturbance, is predictive of long-term hypnotic treatment. Our results imply the limitation of the effectiveness of hypnotic treatment alone for chronic insomnia.

## Introduction

Insomnia is a common disorder with a remarkably high prevalence [1]–[3]. It has been reported that one-fifth of the general population in Japan has symptoms associated with insomnia [4]. Chronic insomnia is known to be associated with subjective daytime fatigue, low energy, difficulties in cognitive performance, and deteriorated quality of life [5]. The disorder has also been known to be a risk factor for the development of somatic diseases such as hypertension and diabetes mellitus [6]–[8]. Furthermore, chronic insomnia is suspected as one of the risk factors for the development of psychiatric disorders, particularly depression and anxiety disorders [9], [10]. Thus, establishment of a better treatment strategy for achieving sufficient improvement of the disorder is desirable.

Benzodiazepines (BZDs) or benzodiazepine receptor agonists (BzRAs) have long been accepted as one of the important treatment choices for insomnia. However, the disadvantages of the long-term use of these kinds of hypnotics, such as the risk of tolerance [11] and dependence [12], have been indicated. Based on this, clinical guidelines of the American Academy of Sleep Medicine suggested that long-term hypnotic treatment can be indicated only for the patients with severe or refractory insomnia or chronic illness [13]. It is also suggested that BZDs should only be used for a short-term period of up to 4 weeks to prevent the occurrence of the disadvantages associated with long-term use [14]. However, there are considerably large numbers of patients with chronic insomnia who require long-term medication with hypnotics due to poor response to treatment [15].

To avoid the long-term use of BZDs or BzRAs as hypnotics, it is necessary to highlight the factors associated with long-term treatment with these types of drugs. However, thus far, there have been apparently no studies to clarify this issue. We therefore investigated the factors associated with long-term treatment with hypnotics among patients with chronic insomnia in order to contribute to the development of an effective treatment strategy for this disorder.

## Materials and Methods

This study was approved by the Ethics Committee of the Neuropsychiatric Research Institute, Tokyo, Japan, and written informed consent was obtained from all the enrolled patients.

Among the consecutive patients who visited the outpatient clinic of the Japan Somnology Center seeking treatment of their sleep problems from May 2003 to December 2009, the subjects for this study were selected from the patients who met the diagnostic criteria for primary insomnia according to the Diagnostic and Statistical Manual of Mental Disorders Fourth Edition Text Revision (DSM-IV-TR). The diagnosis of primary insomnia for all selected patients was confirmed by at least 2 psychiatrists who specialize in sleep disorders. None of the patients had or were previously affected with any other psychiatric disorders, such as major depression, anxiety disorders, or substance abuse. Patients who received antidepressants, antipsychotics, or more than 2 types or unusually high doses of hypnotics were excluded from the study. In cases of the suspected presence of other sleep disorders, 1 night polysomnography and more than 2 weeks of sleep logs were recorded for making differential diagnoses. As a result, 140 consecutive patients with primary insomnia, who met the criteria of chronic insomnia with persistence of insomnia symptoms for at least 6 months [16], were enrolled in this study (68 men and 72 women; mean age, 53.8±10.8 years).

All these patients filled out a self-assessment questionnaire before starting the treatment with the usual dose of BZD or BzRA hypnotic. The questionnaire requested information regarding sex, age at the time of investigation, age at the onset of subjective insomnia, duration of morbidity, marital status (married/unmarried), occupation (employed/unemployed), and educational background (college educated/not). The Pittsburgh Sleep Quality Index (PSQI) was self-assessed for estimating the severity of their insomnia symptoms before starting the treatment [17], [18]. The PSQI included the following sub-items: evaluating sleep quality (C1), sleep latency (C2), sleep duration (C3), habitual sleep efficiency (C4), frequency of sleep disturbance (C5), use of sleeping medication (C6), and daytime dysfunction (C7). The scores of these sub-items range from 0 (no difficulty) to 3 (severe difficulty) and are summed to produce a global measure of sleep disturbance, with a higher score denoting poorer sleep quality (range: 0–21). Simultaneously, all patients completed the Zung Self-Rating Depression Scale (SDS) [19].

At the start of hypnotic treatment for each patient, sleep specialist physicians set a goal to finish the treatment within 6 months since increasing occurrence of dependence and withdrawal symptoms of BZDs or BzRAs are reportedly likely to increase over 6 months of consecutive medication [20], [21]. Based on this, sleep specialist physicians instructed the subject patients to reduce the amount of hypnotics by one-quarter tablet during the treatment period, if their insomnia symptoms improved sufficiently. During the treatment period, all patients visited the outpatient clinic once per month regularly to receive a prescription of BZD or BzRA together with general sleep hygiene education [22]; none of them received any psychotherapy, including cognitive behavioral therapy (CBT).

We divided the patients into 2 groups, namely, those who achieved sufficient improvement of symptoms resulting in the discontinuation of BZD or BzRA medication within a 6-month treatment period (discontinuation group), and those who continued to be treated with those hypnotics even at the end of the treatment period (long-term use group). PSQI was self-assessed at two time points by all the subject patients. In the discontinuation group, PSQI was re-evaluated at the time of cessation of treatment with hypnotics to confirm a sufficient improvement; in the long-term use group, PSQI was re-evaluated 6 months after the start of the treatment.

### Statistical analysis

The Mann-Whitney U test and chi-square test were used for comparison of descriptive variables between the discontinuation group and the long-term use group. The Mann-Whitney U test was also used for the comparison between the 2 groups of the changes in PSQI total and sub-item scores from the baseline to the end of the treatment period. The Wilcoxon signed rank test was used for the comparison of PSQI total and sub-item scores between the baseline and the end of the treatment period.

The factors associated with the discontinuation group were examined by logistic regression analyses including the above-indicated independent variables (sex, age at the time of investigation, age at onset of insomnia, duration of morbidity, marital status, educational background, occupation, SDS scores, and PSQI scores). All variables were initially examined in univariate models. To control for confounding factors and to determine the main correlates, we then performed multivariate logistic regression analysis for all variables that showed a significant correlation (*p*<0.05) in the univariate models.

Receiver operating characteristic (ROC) curves [23] were plotted and the mean estimated area under the curve (AUC) with 95% confidence interval (CI) for the PSQI score at the baseline was calculated for predicting discontinuation of hypnotics. When the slope of the tangent line of the ROC curve was statistically equal to 1 (i.e., AUC = 0.5), the ROC curve was regarded as inaccurate for prediction. The best cut-off value for predicting the discontinuation of hypnotics was determined on the basis of sensitivity, specificity, and positive likelihood ratio (LR+) and negative likelihood ratio (LR−). According to an established method [24], the cut-off value was assessed as adequate when LR+ was 2.0 or higher and LR− was 0.5 or lower.

SPSS version 11.5.1J software for Windows (SPSS Inc., Tokyo) was used for the above statistical analyses. A *p*-value of less than 0.05 was considered to indicate a statistically significant difference.

## Results

Patients' characteristics are shown in Table 1. For the total number of patients (n = 140), the mean age at onset was 50.8±11.0 (mean±SD) years, the mean age at the time of investigation was 53.8±10.8 years, the mean duration of self-reported insomnia morbidity was 2.9±2.3 years (all patients indicated longer than 6 months), and the mean SDS score was 39.7±8.9 points. The male/female ratio was 48.6%/51.4%, 72.1% of the patients were married, 34.3% had a college education, and 56.4% were employed (Table 1).

**Table 1:** Comparison of demographic variables between the discontinued group and the long-term use group.

Variable	Total patients (n = 140)	Discontinued (n = 64)	Long-term use (n = 76)	*p*
Age at the time of investigation (years)	53.8±10.8	53.3±10.5	54.1±11.1	ns
Age at the onset of insomnia (years)	50.8±11.0	50.3±10.9	51.3±11.0	ns
Sex (male:female)	68∶72	29∶35	39∶37	ns
Duration of insomnia morbidity (years)	2.91±2.31	3.0±2.4	2.9±0.3	ns
Marital status (married:unmarried)	101∶39	45∶19	56∶20	ns
Educational background (college education:not)	48∶92	16∶48	32∶44	<0.05
Occupation (employed:unemployed)	79∶61	36∶28	38∶38	ns
Half-life of hypnotic (ultra-short/short/intermediate/long)	(49/64/19/8)	(23/32/7/2)	(26/32/12/6)	ns
Dose of hypnotic (mg/day in diazepam equivalents)	6.0±2.2	6.1±2.2	5.9±2.1	ns
SDS score (points)	39.70±8.86	41.1±9.9	38.5±7.8	ns
PSQI total score (points)	13.6±2.0	12.3±1.8	14.8±1.4	<0.01

Values are expressed as means ±SD. The Mann-Whitney U test was used for the comparison of continuous variables between the 2 groups as follows: age, duration of insomnia morbidity, dose of hypnotics, and SDS and PSQI scores. The chi-square test was used for the comparison of categorical variables between the 2 groups as follows: sex, marital status, educational background, occupation, and half-life of hypnotic.

ns =  not significant; SDS =  Zung Self-Rating Depression Scale; PSQI =  Pittsburgh Sleep Quality Index.

Of all the patients, 35.0% received an ultrashort elimination half-life BZD or BzRA, 45.7% received a short elimination half-life BZD, 13.6% received an intermediate elimination half-life BZD, and 5.7% received a long elimination half-life BZD. The amount of BZD or BzRA manifested as diazepam equivalent doses was 6.1±2.2 mg in the discontinued group and 5.9±2.1 mg in the long-term use group.

There were 64 patients (45.4%) in the discontinuation group, and the remaining 76 patients (54.6%) were in the long-term use group. Among the long-term use group, 28 patients continued to receive the same dose of BZD or BzRA as the baseline dose, 41 were prescribed a higher dose of the same BZD or BzRA than the baseline dose, or other types of BZDs or BzRAs additionally because of the ineffectiveness of treatment, and 7 were prescribed different types of BZDs or BzRAs during the 6-month treatment period.

In the comparison of demographic variables, there were significant differences between the discontinuation group and the long-term use group regarding the educational background (*p*<0.05) and the PSQI score at the baseline (*p*<0.01). No significant differences were found between the 2 groups in terms of age at the time of the investigation, age at the self-reported onset of insomnia, duration of insomnia morbidity, SDS score, marital status, occupation, half-life of BZD or BzRA, and diazepam equivalent doses of these hypnotics (Table 1).

For all patients, the mean PSQI total score at the baseline was 13.6±2.0 points and the score at the end of the treatment period was 9.3±2.5 points. The results of comparison of PSQI total and sub-item scores between the baseline and the end of the treatment period are shown in Table 2. At the second assessment of the PSQI, the C1 (sleep quality) to the C5 (frequency of sleep disturbance) scores decreased both in the discontinuation group and the long-term use group, the C6 (use of sleeping medication) score was increased in both groups, and the C7 (daytime dysfunction) score was decreased in the discontinuation group whereas it was increased in the long-term use group. There were significant differences in the changes in the PSQI total score as well as in the C2 (sleep latency), C5 (frequency of sleep disturbance), C6 (use of sleeping medication), and C7 (daytime function) scores from the baseline to the end of the treatment period between the discontinuation group and the long-term use group (total scores, C2, C5, and C7, *p*<0.01; C6 score, *p*<0.05), whereas no significant differences in the C1 (sleep quality), C3 (sleep duration), and C4 (habitual sleep efficiency) scores between the 2 groups were found (Table 2).

**Table 2:** Comparison of PSQI total and sub-item scores between the baseline and the end of the treatment period, and comparison of changes in these scores between the discontinued group and the long-term use group from the baseline to the end of the treatment period.

	Total patientsa) (n = 140)		Discontinueda) (n = 64)		Long-term usea) (n = 76)		Change in scores between the 2 time pointsb)	
	Baseline	End of follow-up	Baseline	End of follow-up	Baseline	End of follow-up	Discontinued	Long-term use
PSQI total score	13.6±2.0	9.3±2.5	12.3±1.8	6.9±1.2	14.8±1.4	11.2±1.3	5.4±2.0	3.6±2.0*
C1: sleep quality	2.2±0.6	1.5±0.7	1.9±0.6	1.0±0.6	2.6±0.5	1.9±0.5	0.8±0.8	0.7±0.7
C2: sleep latency	2.5±0.6	1.1±0.5	2.6±0.6	0.9±0.3	2.4±0.5	1.3±0.5	1.6±0.7	1.1±0.7*
C3: sleep duration	2.3±0.5	1.2±0.4	2.2±0.5	1.1±0.2	2.4±0.5	1.4±0.5	1.1±0.6	1.0±0.6
C4: habitual sleep efficiency	2.1±0.6	1.4±0.6	1.8±0.6	1.0±0.3	2.4±0.5	1.8±0.6	0.8±0.6	0.7±0.8
C5: sleep disturbance	2.2±0.6	1.4±0.6	2.2±0.7	1.0±0.0	2.2±0.5	1.8±0.6	1.2±0.7	0.5±0.7
C6: use of sleeping medication	1.8±1.1	2.4±0.8	1.1±1.0	1.9±0.8	2.4±0.8	2.7±0.5	−0.8±1.3	−0.3±1.1
C7: daytime dysfunction	0.5±0.6	0.3±0.5	0.7±0.6	0.0±0.1	0.4±0.6	0.5±0.6	0.6±0.6	−0.1±0.6

a) The Wilcoxon signed rank test was used for comparison of the scores between the 2 time points.

b) The Mann-Whitney U test was used for comparison of the changes in these scores between the 2 groups.

Values are expressed as means ±SD for continuous variables.

*p<0.01;

**p<0.05; PSQI =  Pittsburgh Sleep Quality Index.

Logistic regression analyses on the associated factors for the long-term use group were performed with the following 8 explanatory variables: sex, age at the self-reported onset of insomnia, age at the time of investigation, duration of the insomnia morbidity, educational background, marital status, SDS score, and PSQI total score. Among these, sex, educational background, occupation, and marital status were treated as categorical variables, and the other measured items were treated as continuous variables. As a result, multivariate analysis revealed that the long-term use of hypnotics was significantly associated only with the increase in the total PSQI score (OR = 2.8, 95% CI = 2.1–26.9, *p*<0.01) (Table 3).

**Table 3:** Logistic regression analysis of the associated factors for the long-term use of hypnotics (n = 140).

	Univariate odds ratio (95% CI)	*p*	Multivariate odds ratio (95% CI)	*p*
Sex (male/female)		ns		ns
Age at the time of investigation (years)		ns		ns
Age at onset (years)		ns		ns
Duration of morbidity (years)		ns		ns
Marital status (married/unmarried)		ns		ns
Educational background (college educated/not)	2.2 (1.1–4.5)	<0.05		ns
Occupation (employed/unemployed)		ns		ns
Half-life of hypnotic (ultrashort/short/intermediate/long)		ns		ns
SDS score (points)		ns		ns
PSQI total score (points)	2.8 (2.0–3.99)	<0.01	2.8 (2.0–4.0)	<0.01

CI denotes confidence intervals.

ns =  not significant; PSQI =  Pittsburgh Sleep Quality Index; SDS =  Zung Self-Rating Depression Scale.

**Figure 1:**
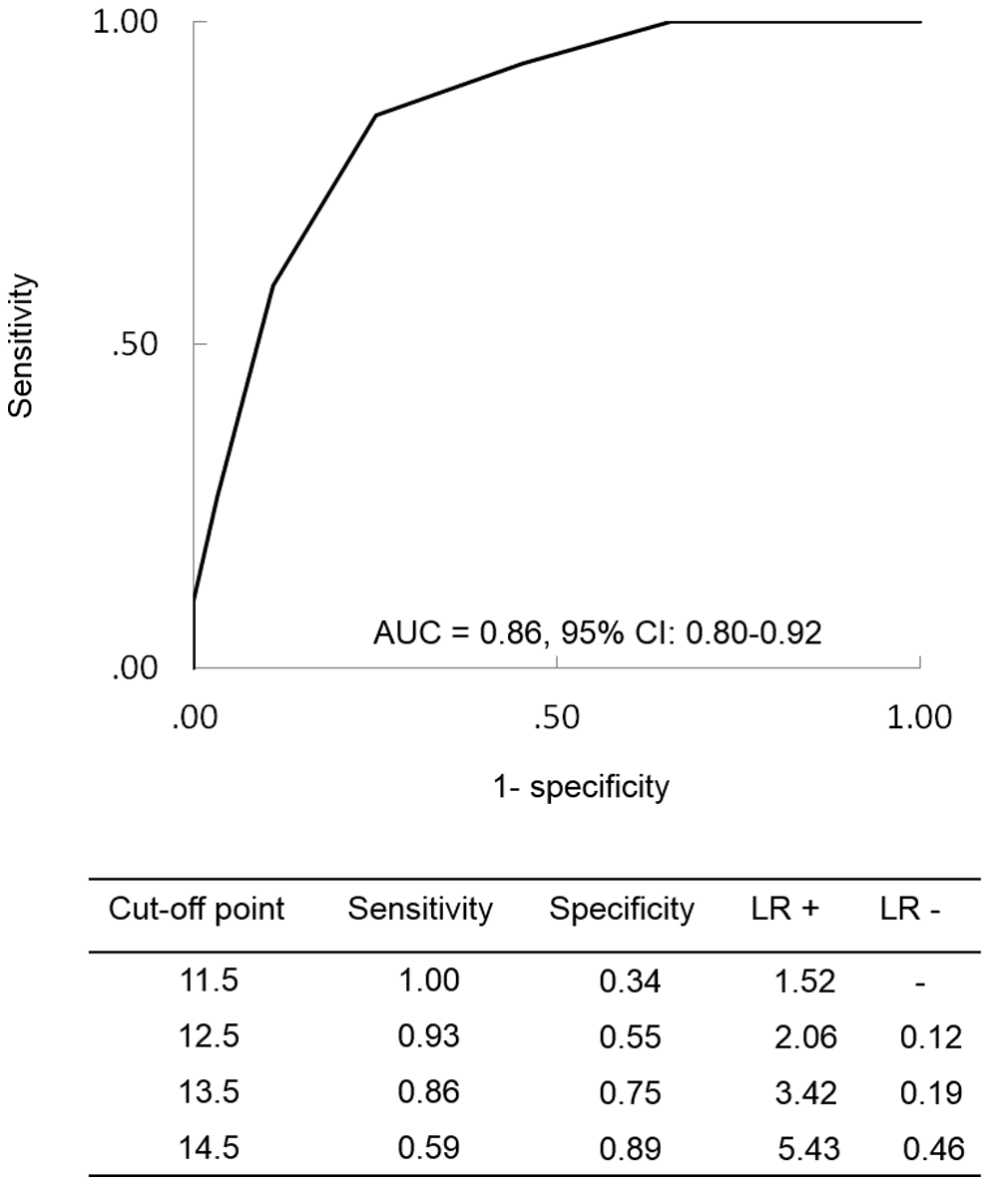
Predictive cut-off point of the Pittsburgh Sleep Quality Index for the long-term use of hypnotics estimated with the receiver operating characteristic (ROC) curve. CI denotes confidence intervals. AUC =  area under the curve.

We created the ROC curve to examine the cut-off PSQI total score at the baseline for predicting discontinuation of hypnotic treatment within a 6-month period. The AUC of the ROC curve was 0.86 (95% CI = 0.80–0.92). The cut-off total PSQI score at the baseline for predicting the discontinuation of hypnotic treatment within 6 months was estimated at 13.5 points; this cut-off value had a sensitivity of 0.86, a specificity of 0.75, LR+ = 3.42, and LR− = 0.19 (Figure 1).

Logistic regression analysis was conducted again to clarify the associated factors for the long-term use of hypnotics among the sub-item scores of the PSQI. Logistic regression analysis was performed with 7 sub-item scores of PSQI {sleep quality (C1), sleep latency (C2), sleep duration (C3), habitual sleep efficiency (C4), frequency of sleep disturbance (C5), use of sleeping medication (C6), and daytime dysfunction (C7)} as independent variables. The sub-item scores were treated as continuous variables. The multivariate analysis revealed that the long-term use of hypnotics for the treatment of insomnia was associated with an increase in the C1 (OR = 8.4, 95% CI = 2.4–30.0, *p*<0.01), C3 (OR = 3.6, 95% CI = 1.1–11.5, *p*<0.05), C4 (OR = 11.1, 95% CI = 3.6–33.9, *p*<0.01), and C6 (OR = 3.4, 95% CI = 1.9–6.2, *p*<0.01) scores (Table 4).

**Table 4:** Logistic regression analysis of the associated factors for discontinuation of hypnotics using PSQI sub-item scores as explanatory variables.

PSQI sub-item	Univariate odds ratio (95% CI)	*p*	Multivariate odds ratio (95% CI)	*p*
C1: sleep quality	12.1 (4.9–29.5)	<0.01	8.4 (2.4–30.0)	<0.01
C2: sleep latency		ns		ns
C3: sleep duration	2.2 (1.1–4.4)	<0.05	3.6 (1.1–11.5)	<0.05
C4: habitual sleep efficiency	8.7 (3.7–20.5)	<0.01	11.1 (3.6–33.9)	<0.01
C5: sleep disturbance		ns		ns
C6: use of sleeping medication	3.9 (2.5–6.0)	<0.01	3.4 (1.9–6.2)	<0.01
C7: daytime dysfunction	0.56 (0.3–1.0)	<0.05		ns

CI denotes confidence intervals.

ns =  not significant; PSQI =  Pittsburgh Sleep Quality Index.

## Discussion

In this study, the ratio of chronic insomnia patients who needed to continue BZD or BzRA medication for a 6-month treatment period reached more than half (54.6%) of the total number of patients enrolled in this study, which is similar to that previously reported [15]. Thus, approximately half of the patients with chronic insomnia are assumed to become long-term users of BZD or BzRA medication. As for the demographic backgrounds of the patients in this study, there were no remarkable differences in age, sex [4], duration of insomnia [15], and severity of depression [25] compared with general insomnia patients. However, the PSQI total score of the patients in this study was relatively higher than that of the insomnia patients in previous studies [26], [27]. For this reason, it was speculated that patients with severe insomnia are likely to visit our clinic since our clinic is a specialized and referral-based sleep disorder center. Given this fact, our results strongly indicate that it is difficult to sufficiently improve the symptoms of chronic insomnia, particularly in severe cases, with the usual dose of a single type of BZD or BzRA [28].

The results of the present logistic regression analysis showed that only the PSQI total score, not the demographic factors, appeared as the associated factor for the long-term use of the treatment. Significant relationships of female sex and older age to insomnia morbidity have been reported [4]. However, the result of the multivariate regression analysis in this study showed no significant association between long-term use of hypnotics and these demographic factors. The reason for this discrepancy is unclear. However, this result implies that there may be a difference between the factors for vulnerability to morbidity and those for responsiveness to the treatment of insomnia.

Our results showed that almost half of the patients (n = 68) had an abnormally high SDS score, although we excluded the patients affected with major depression based on the diagnostic criteria of DSM-IV-TR through the clinical interviews. However, most of them remained in the range of mild depression [19]. Thus, it seems likely that their depressive symptoms were caused secondarily by the insomnia symptom [29], although we did not evaluate the changes in depressive symptoms during the treatment period. Additionally, as shown in the results of the logistic regression analysis, the depressive symptom was not associated with the response to hypnotic treatment in this population with chronic insomnia.

The ROC curve notably revealed that the cut-off value for predicting patient discontinuation of hypnotic treatment was a PSQI score of 13.5 points at the baseline. This PSQI score was relatively higher than that of the general insomnia population at the clinical setting (average score: 8.9–10.4 points) [17], [18]. Thus, although our sample population was assumed to have a sampling bias as mentioned above, our results suggest that the symptoms of patients with severe chronic insomnia having a high PSQI score are difficult to treat by a single type of BZD or BzRA at the usual dose.

Among the sub-items of the PSQI, there was a significant association of long-term use of hypnotics with C6 (sleeping medication). Considering that a high score of C6 means high frequency of hypnotic medication at the baseline in the present study, it is strongly suspected that they already had treatment resistance or tolerance to preceding BZD or BzRA medication. Interestingly, in the present study, long-term use of hypnotics showed significant association with C1 (sleep quality), C3 (sleep duration), and C4 (habitual sleep efficiency), all of which are constituents of the symptoms of sleep maintenance insomnia, whereas C2 (sleep latency), which is clearly related to sleep initiation disturbance [18], did not show statistical association with the long-term use. In addition, the results of the logistic regression analysis showed that the half-life of BZD or BzRA was not associated with the long-term use of hypnotics. Taking these results together, sleep initiation insomnia can be substantially improved with hypnotics of any elimination half-life, as shown in previous reports [30]–[32]. On the other hand, sleep maintenance insomnia has been suggested to be difficult to improve only by treatment with hypnotics [33]. The results of long-term use of hypnotic treatment in patients with a higher C1 (sleep quality), C3 (sleep duration), or C4 (habitual sleep efficiency) score in the present study could support this hypothesis.

This study has several limitations. Firstly, although we showed self-reported duration of insomnia morbidity in the subject patients, we do not have sufficient information about the content, regularity, and duration of previous treatment. Secondly, because this study was conducted on patients in a single sleep disorder center, a sampling bias as indicated above should be considered. In fact, the PSQI total score in the patients examined in this study (13.6 points) was relatively higher than the score previously reported for the general insomnia population (average score: 8.9 to 10.4 points) [17], [18]. Thirdly, although the treatment refractoriness was thought to be the main reason for the long-term use of BZDs or BzRAs, the other reason (dependence on drug or blind faith in the benefit of hypnotic treatment of the subject patients) should also be considered for this phenomenon. However, details about the patient's attitude about hypnotic treatment could not be obtained in the present study. Fourthly, hypnotic treatment in this study was conducted with an open and uncontrolled design, and therefore a randomized and controlled trial would be necessary in the future to investigate the factors associated with long-term use of BZDs or BzRAs in patients with chronic insomnia. Finally, although we evaluated the treatment response of patients only at the end of a 6-month treatment period, the short-term evaluation of symptoms as well as the longitudinal comparison of symptoms throughout the study period would be necessary to clarify details of the factors associated with the long-term use of BZD or BzRA hypnotics.

In conclusion, the results of this study revealed that the long-term treatment with BZD or BzRA hypnotics is associated with the severity of insomnia symptoms, and that insomnia patients whose PSQI scores are 13.5 points or higher are likely to become resistant to long-term hypnotic treatment. In addition, it appears difficult to sufficiently improve sleep maintenance insomnia with the usual dose of a single type of BZD or BzRA hypnotic. CBT has been reported to be effective for patients with chronic insomnia having hypnotic dependency [34] and has become one of the important treatment alternatives when tapering the dose of hypnotic medication in the chronic treatment of refractory insomnia [35]. To prevent the long-term use of BZD or BzRA hypnotics, adjunctive therapy with CBT should be considered for patients with treatment-resistant insomnia [13].
